# Kalman Filter Implementation of Subglottal Impedance-Based Inverse Filtering to Estimate Glottal Airflow during Phonation

**DOI:** 10.3390/app12010401

**Published:** 2021-12-31

**Authors:** Juan P. Cortés, Gabriel A. Alzamendi, Alejandro J. Weinstein, Juan I. Yuz, Víctor M. Espinoza, Daryush D. Mehta, Robert E. Hillman, Matías Zañartu

**Affiliations:** 1 Department of Electronic Engineering, Universidad Técnica Federico Santa María, Valparaiso 2390123, Chile; 2 Institute for Research and Development on Bioengineering and Bioinformatics, Consejo Nacional de Investigaciones Científicas y Técnicas–Universidad Nacional de Entre Ríos, Oro Verde 3100, Argentina; 3 Department of Biomedical Engineering, Universidad de Valparaíso, Valparaiso 2362905, Chile; 4 Department of Sound, Universidad de Chile, Santiago 8340380, Chile; 5 Massachusetts General Hospital, Boston, MA 02114, USA; 6 Speech and Hearing Bioscience and Technology Program, Harvard Medical School, Boston, MA 02115, USA; 7 MGH Institute of Health Professions, Boston, MA 02129, USA

**Keywords:** vocal hyperfunction, inverse filtering, Kalman filter

## Abstract

Subglottal Impedance-Based Inverse Filtering (IBIF) allows for the continuous, non-invasive estimation of glottal airflow from a surface accelerometer placed over the anterior neck skin below the larynx. It has been shown to be advantageous for the ambulatory monitoring of vocal function, specifically in the use of high-order statistics to understand long-term vocal behavior. However, during long-term ambulatory recordings over several days, conditions may drift from the laboratory environment where the IBIF parameters were initially estimated due to sensor positioning, skin attachment, or temperature, among other factors. Observation uncertainties and model mismatch may result in significant deviations in the glottal airflow estimates; unfortunately, they are very difficult to quantify in ambulatory conditions due to a lack of a reference signal. To address this issue, we propose a Kalman filter implementation of the IBIF filter, which allows for both estimating the model uncertainty and adapting the airflow estimates to correct for signal deviations. One-way analysis of variance (ANOVA) results from laboratory experiments using the Rainbow Passage indicate an improvement using the modified Kalman filter on amplitude-based measures for phonotraumatic vocal hyperfunction (PVH) subjects compared to the standard IBIF; the latter showing a statistically difference (*p*-value = 0.02, *F* = 4.1) with respect to a reference glottal volume velocity signal estimated from a single notch filter used here as ground-truth in this work. In contrast, maximum flow declination rates from subjects with vocal phonotrauma exhibit a small but statistically difference between the ground-truth signal and the modified Kalman filter when using one-way ANOVA (*p*-value = 0.04, *F* = 3.3). Other measures did not have significant differences with either the modified Kalman filter or IBIF compared to ground-truth, with the exception of H1–H2, whose performance deteriorates for both methods. Overall, both methods (modified Kalman filter and IBIF) show similar glottal airflow measures, with the advantage of the modified Kalman filter to improve amplitude estimation. Moreover, Kalman filter deviations from the IBIF output airflow might suggest a better representation of some fine details in the ground-truth glottal airflow signal. Other applications may take more advantage from the adaptation offered by the modified Kalman filter implementation.

## 1. Introduction

Voice disorders are a health problem of significant concern in our society. In the United States, voice disorders affect about 7% of the working population [1–4]. Many of these voice disorders are chronic or recurring conditions that result from repeated detrimental patterns of vocal behavior, referred to as vocal hyperfunction (VH) [5,6]. Subtypes of VH include phonotraumatic VH (PVH) that is associated with the formation of benign vocal fold lesions (e.g., nodules) due to phonotrauma, and non-phonotraumatic VH that is associated with the dis-coordination of laryngeal muscle control in the absence of structural abnormalities (often diagnosed as primary muscle tension dysphonia) [7]. Despite the significant prevalence of these disorders, very little is known about the underlying physical mechanisms of VH. Given that multiple factors contribute and interact in different ways to cause and sustain VH disorders, there are non-specific, broad-based behavioral treatments that are inefficient, make patient compliance more challenging, and make it difficult or impossible to link improvements in vocal function to specific parts of the therapy program [8].

Several efforts have been carried out to develop objective measures that can capture VH, such as aerodynamic measures obtained from estimates of the glottal airflow [5,9,10], relative fundamental frequency [11], estimates of spectral tilt of the voice source [12,13], and cepstral-related measures [14], among others. However, these measures are typically applied in the context of a laboratory assessment with sustained vowels and do not capture the nuances of VH in natural speech during daily activities.

The objective assessment of VH is expected to be significantly enhanced through ambulatory monitoring of vocal function. Ambulatory voice monitoring aims at providing complementary information that current clinical methods cannot offer, such as long-term behavior through the use of high-order statistics [14–20]. An ambulatory approach that could precisely pinpoint the instance, duration, and type of VH behavior would have the capability to provide transformative advancements in how clinical practices monitor, evaluate, and treat VH. Efforts in ambulatory methods are heading in this direction [17–19], but there are many associated challenges.

Some of the ambulatory voice monitors use either a microphone signal to estimate fundamental frequency (*f*_0_) and jitter [21], a surface electromyograph to estimate increased muscle tension [22], or a neck-surface accelerometer over the extrathoracic trachea to estimate sound pressure level, fundamental frequency, voicing activity, vocal dose, and related measures[15,23–28], as well as aerodynamic, cepstral and related parameters [14,16]. Aerodynamic measures have been successfully used to differentiate both phonotraumatic and non-phonotraumatic VH patients from matched controls using sustained vowels [9,29], and have been shown to become salient features of compensatory mechanisms associated with VH in modeling studies [30,31]. Thus, these aerodynamic measures have a strong potential to enhance the ability to identify VH in ambulatory settings [16].

Given that traditional assessment of aerodynamic signals using a Rothenberg mask [32] is not feasible for ambulatory scenarios, indirect estimation methods are required. The Subglottal Impedance-Based Inverse Filtering (IBIF) scheme [33] allows for estimating the glottal airflow signal from neck-surface vibration. The IBIF approach was recently tested in a discrimination task using week-long ambulatory recordings for 50 patients with vocal fold nodules and 50 matched healthy-control subjects [16]. The results of classification task using aerodynamic features outperformed previous efforts with other measures [14,15,17,19] and provides a new avenue to improve the assessment and treatment of VH disorders.

Despite of these advances, unquantified uncertainties are associated to the estimation of the glottal airflow signal with the IBIF scheme due to a number of factors. First, the determination of the IBIF model parameters uses inverse filtering of the oral airflow from few sustained vowel samples, which can lead to IBIF parameter variations for different vowels and pitch conditions [16,34]. The latter becomes especially challenging for high-pitched female voices, which are common in ambulatory studies. In addition, there are combined measurement uncertainties from the accelerometer due to sensor positioning, skin attachment, temperature, etc. Furthermore, there is no direct reference that can be used to quantify these combined effects in ambulatory scenarios. Thus, there is a need to quantify the magnitude of the uncertainty in the estimation process, and to potentially improve the estimation of the aerodynamic signals through the IBIF framework.

To address the aforementioned limitations, we propose a Kalman filter (KF) implementation of the IBIF filter, which allows for both assessing the estimation uncertainty and correcting for potential deviations in the airflow signal estimates. The KF structure is based in a Moving Average (MA) Kalman Filter with colored state noise modeling the glottal airflow signal. To assess the accuracy of the KF, we compare aerodynamic measures describing the glottal airflow signal obtained from the oral mask using a notch-filter [35,36], the standard IBIF [33], and the modified Kalman filter for a group of PVH and healthy-controls subjects reading a phonetically balanced passage.

The paper is structured as follows: In Section 2, we present the methods utilized to estimate glottal airflow, namely the IBIF method and its Kalman filter implementation. Then, in Section 3, we describe the experimental setup with participants with PVH and vocally healthy control subjects. In Section 4, we present the results of the experiments, and in Section 5, we discuss them in detail. Finally, in Section 6, we present the conclusions and future work.

## 2. Materials and Methods

### 2.1. Standard IBIF Implementation

The IBIF scheme is described in the frequency domain, where the glottal airflow (also referred to as glottal volume velocity, GVV) and the acceleration signal are related by

(1) $${\dot{U}}_{\text{skin}}(\omega )=T_{\text{skin}}(\omega )\cdot U_{sub}(\omega )$$

where ${\dot{U}}_{\text{skin}}(\omega )$ is the acceleration signal, *U*_*sub*_(*ω*) is the inverted GVV (assuming source is a dipole, that is, two equal and opposite volume velocity sources [37]), and *T*_*skin*_(*ω*) is the neck-skin frequency response. In what follows, we remove the frequency dependency *ω* in the expressions for the sake of clarity. *T*_*skin*_ can be modeled by:

(2) $$T_{\text{skin}\;}=\frac{{\dot{U}}_{\text{skin}\;}}{U_{sub\;}}=\frac{H_{sub1}\cdot Z_{sub\text{2 }}\cdot H_{d}}{Z_{sub2}+Z_{\text{skin}}},$$

where *H*_*sub*1_ is the frequency response of the subglottal section from the glottis to the accelerometer location, and *H*_*d*_ = *jω* is a derivative filter (similar to the lip radiation effect, except that in this case is the acceleration in free field). *Z*_*sub*2_ is a frequency-dependent driving-point impedance corresponding to the subglottal section [38] below the accelerometer position. *Z*_*skin*_ is the neck-skin impedance modeled as a mechanical analog of a resistor-inductor-capacitor circuit in series:

(3) $$Z_{\text{skin}}=R_{m}+j\left(\omega M_{m}-\frac{K_{m}}{\omega }\right)+\frac{j\omega M_{acc}}{A_{acc}},$$

where *R*_*m*_, *M*_*m*_, and *K*_*m*_ are the per-unit-area resistance, inertance, and stiffness of the skin, respectively. The radiation impedance due to the accelerometer loading is modeled as a derivative term *jω* times the mass *M*_*acc*_ divided by the surface *A*_*acc*_ (per-unit-area) of the accelerometer and the coating or mounting disk attached to it [39]. These parameters are subject specific, and therefore involve calibration factors that can be fitted per subject using a reference GVV signal and an optimization method. The calibration factors ***Q*** = {*Q*_*i*_}_*i*=1,…,5_ are defined as:

(4) $$Q=\left\{Q_{1},Q_{2},Q_{3},Q_{4},Q_{5}\right\}$$

(5) $$R_{m}=2320\cdot Q_{1}\;\left[\text{g}\cdot {\text{s}}^{-1}\cdot {\text{cm}}^{-2}\right],$$

(6) $$M_{m}=2.4\cdot Q_{2}\;\left[\text{g}\cdot {\text{cm}}^{-2}\right],$$

(7) $$K_{m}=491,000\cdot Q_{3}\;\left[\text{dyn}\cdot {\text{cm}}^{-3}\right],$$

(8) $$L_{trachea\;}=10\cdot Q_{4}\;[\text{cm}],$$

(9) $$L_{sub1}=5\cdot Q_{5}\;[\text{cm}],$$

where *L*_*trachea*_ (related to the length of the trachea) and *L*_*sub*1_ (related to sensor position on the neck-surface) are embedded in *Z*_*sub*2_ and *H*_*sub*1_, respectively. The derivation of these terms is beyond the scope of this paper and details can be found in [33,40]. Given the ***Q*** factors above, the impulse response of neck-skin *h*(*n*) in the time domain is obtained by first taking the fast Fourier transform (FFT) of *T*_*skin*_(*ω*) with *N* points, which becomes ${\hat{T}}_{\text{skin}\;}(k)$ with *k* = 0, 1, …, *N* − 1, where *N* is the number of FFT frequency points. Then, after forcing ${\hat{T}}_{\text{skin}}(k)$ to be conjugate symmetric $\left({\hat{T}}_{\text{skin}}(k)={\hat{T}}_{\text{skin}}^{*}(N-k)\right)$, we take the inverse FFT to obtain a real impulse response *h*(*n*). In this way, the resulting IBIF filter is implemented as a deterministic finite impulse response filter (FIR) of length *N*. Therefore, in the time domain, the IBIF scheme assumes that the GVV signal *x*(*n*) is convolved with the impulse response *h*(*n*) to produce an output signal *y*(*n*), which corresponds to the neck-surface acceleration. Since we are interested in estimating *x*(*n*), the discrete frequency response ${\hat{T}}_{\text{skin}}(k)$ is inverted to yield ${\tilde{\text{T}}}_{\text{skin}\;}(k)=1/{\hat{T}}_{\text{skin }}(k)$ and, as with ${\hat{T}}_{\text{skin}}(k)$, it is also forced to be conjugate symmetric, so when taking the IFFT the sequence $\tilde{\text{h}}(n)$ is obtained, which is the impulse response of ${\tilde{\text{T}}}_{\text{skin}}(k)$. Therefore, the GVV signal *x*(*n*) can be estimated through the convolution of the acceleration signal *y*(*n*) and the response $\tilde{\text{h}}(n)$. One limitation of this approach is the assumption of fixed ***Q*** factors for each subject. However, these factors contain certain degree of uncertainty [34,41] due to small changes either in the mechanical properties of the neck-skin tissue, as well as changes in the effective length of the trachea when the speaker is voicing in continuous speech. Therefore, a better approach to estimate the GVV signal would be to consider the uncertainty associated to the estimation process with an adaptive filter.

### 2.2. Formulation of IBIF Model Based on a Kalman Filter

Even though the IBIF algorithm performs well in laboratory settings where the calibration procedure is done with a Rothenberg mask, there are uncertainties related to the application of the IBIF filter in ambulatory settings. First, the position and arrangement of the sensor during in field monitoring might not match laboratory specifications, so the subject-specific parameters could change slightly. One approach for tracking relevant latent signals (i.e., GVV) of a given process (i.e., IBIF) based on related noisy/perturbed observations (i.e., neck-skin acceleration) is the use of a Bayesian approach, which allows to simultaneously estimate both the unknown signal and its uncertainty [42]. Under the assumption of linearity and Gaussian distributions for the unknown states, a Kalman Filter is the optimal Bayesian estimator. In this work, we propose an alternative formulation of IBIF combining the state-space framework with the MA canonical form [43] obtained from the *h*(*n*) impulse response:

(10) x(n+1)=Ax(n)+w(n),

(11) y(n)=Cx(n)+v(n).

What follows is the instantiation of a Kalman filter from the model (10) and (11), to our particular problem, where ***x***(*n*) is the state vector containing the GVV signal: ***x***(*n*) = [*x*(*n* − *N* + 1) *x*(*n* − *N* + 2) *x*(*n* − *N* + 3) ⋯ *x*(*n*)]^*T*^ where *N* is the length of the skin-impulse response. Following [43], the transition matrix **A** is given by:

$$A=\left[\begin{array}{cccccc} 0 & 1 & 0 & \cdots & 0 & 0\\ 0 & 0 & 1 & \cdots & 0 & 0\\ \vdots & \vdots & \ddots & \vdots & \vdots & \vdots \\ 0 & 0 & 0 & \cdots & 0 & 1\\ 0 & 0 & 0 & \ldots & 0 & 0 \end{array}\right]\in {\mathbb{R}}^{N\times N},$$

and **w**(*n*) is a Gaussian process noise with zero mean and covariance matrix:

$$R_{w}=\left[\begin{array}{ccccc} 0 & 0 & \ldots & 0 & 0\\ 0 & 0 & \ldots & 0 & 0\\ \vdots & \vdots & \ddots & \vdots & \vdots \\ 0 & 0 & \ldots & 0 & 0\\ 0 & 0 & \ldots & 0 & \sigma_{w}^{2} \end{array}\right]\in {\mathbb{R}}^{N\times N}.$$

The initial condition is specified with the mean **m**_0_ = **E**(**x**_0_) and covariance **P**_0_ = **E**((**x**_0_ − **m**_0_)(**x**_0_ − **m**_0_)^*T*^) of the initial state **x**_0_.

The observation Equation (11) relates the accelerometer signal *y*(*n*) as the convolution between the unobserved state **x** and the neck-skin impulse response *h*(*n*) with coefficients:

$$C={\left[\begin{array}{lllll} h(0) & h(1) & h(2) & \ldots & h(N-1) \end{array}\right]}^{T}\in {\mathbb{R}}^{1\times N}.$$

According to (11), Gaussian measurement noise *v*(*n*) with mean zero and variance $\sigma_{v}^{2}$ is assumed as the additive perturbation to the observed signal. Implementation of the standard MA Kalman filter for a discrete-time set *n* = 1, …, *T* is described in Algorithm 1:


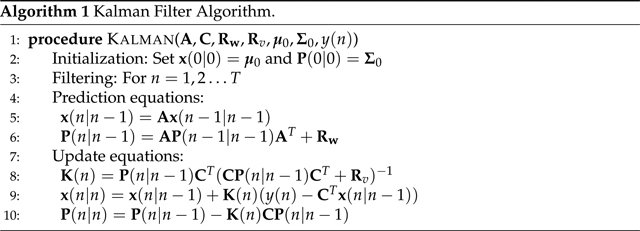


The state matrix A is circular, and the state vector is defined by including the glottal flow for different delays. Therefore, when the filter is applied, states with different delays *n* − *N* + 1, *n* − *N* + 2, …, *n* − 1 are estimated conditioned on the observations up to the current time index *n*, i.e., future information is used in the inference process. In this case, the structure of the Kalman filter in Equations (10) and (11) fulfill that of a fixed-lag smoother [44]. It is important to notice that the canonical MA framework assumes that the GVV signal follows a Gaussian distribution with zero mean and variance $\sigma_{W}^{2}$ (note that the last term in Equation (10) is *x*(*n*) = *w*(*n*), where $w(n)\approx N\left(0,\sigma_{w}^{2}\right)$.) In the following section, we propose a colored noise model that resembles a physiological glottal spectrum in accordance to the source-filter theory of voice production [45].

### 2.3. Glottal Flow Model for the Kalman Filter

According to Fant’s source-filter theory of speech production [45], the glottal excitation is assumed independent of the vocal tract. Even though there is evidence for certain cases of non-linear coupling between the glottal source and the vocal tract [25,40], the source-filter theory has served well for the development of glottal source modeling and estimation. In terms of modeling the glottal source, parametric time domain models have been proposed, such as the Rosenberg model of glottal pulse [46] and the Lijecrants-Fant (LF) model of the derivative of the glottal pulse [47]. These models are widely used and serve as templates to other more complex source modeling strategies [48,49]. In this work, we use the Rosenberg model to construct a glottal spectrum, due to its efficacy in modeling colored noise as a low-pass filter with fewer parameters than the LF model [50,51].

Rosenberg Model for the Glottal Pulse

A parametric model of the glottal pulse can be obtained from the Rosenberg model [46], which can be formulated as [51]:

$$g[n]=\{\begin{array}{ll} 0.5\left[1-\text{cos}(\pi (n+1))/N_{1}\right], & 0\leq n\leq N_{1}-1,\\ \text{cos}\left(0.5\pi \left(n+1-N_{1}\right)/N_{2}\right), & N_{1}\leq n\leq N_{1}+N_{2}-1,\\ 0, & \text{otherwise}, \end{array}$$

where *N*_1_ is the number of samples of the opening phase and *N*_2_ is the number of samples of the closing phase. For a sequence of 96 samples (equivalent to approx. 210 Hz fundamental frequency, pitch period of 4.8 msec., and sampling frequency *f*_*s*_ = 20 kHz), with *N*_1_ = 25 and *N*_2_ = 10, the *z*-transform *G*(*z*) has the form:

(12) $$G(z)=z^{-33}\prod_{k=1}^{33}\left(-b_{k}^{-1}\right)\prod_{k=1}^{33}\left(1-b_{k}z\right),$$

where *b*_*k*_ corresponds to the zeros of *G*(*z*), which can also be written in the following form:

(13) $$\begin{array}{l} G(z)=g[0]+g[1]z^{-1}+g[2]z^{-2}+\cdots +g[N-1]z^{-(N-1)},\\ =\beta_{0}+\beta_{1}z^{-1}+\beta_{2}z^{-2}+\cdots +\beta_{N-1}z^{-(N-1)},\\ =\sum_{k=0}^{N-1}\beta_{k}z^{-k}. \end{array}$$

**Figure 1.**
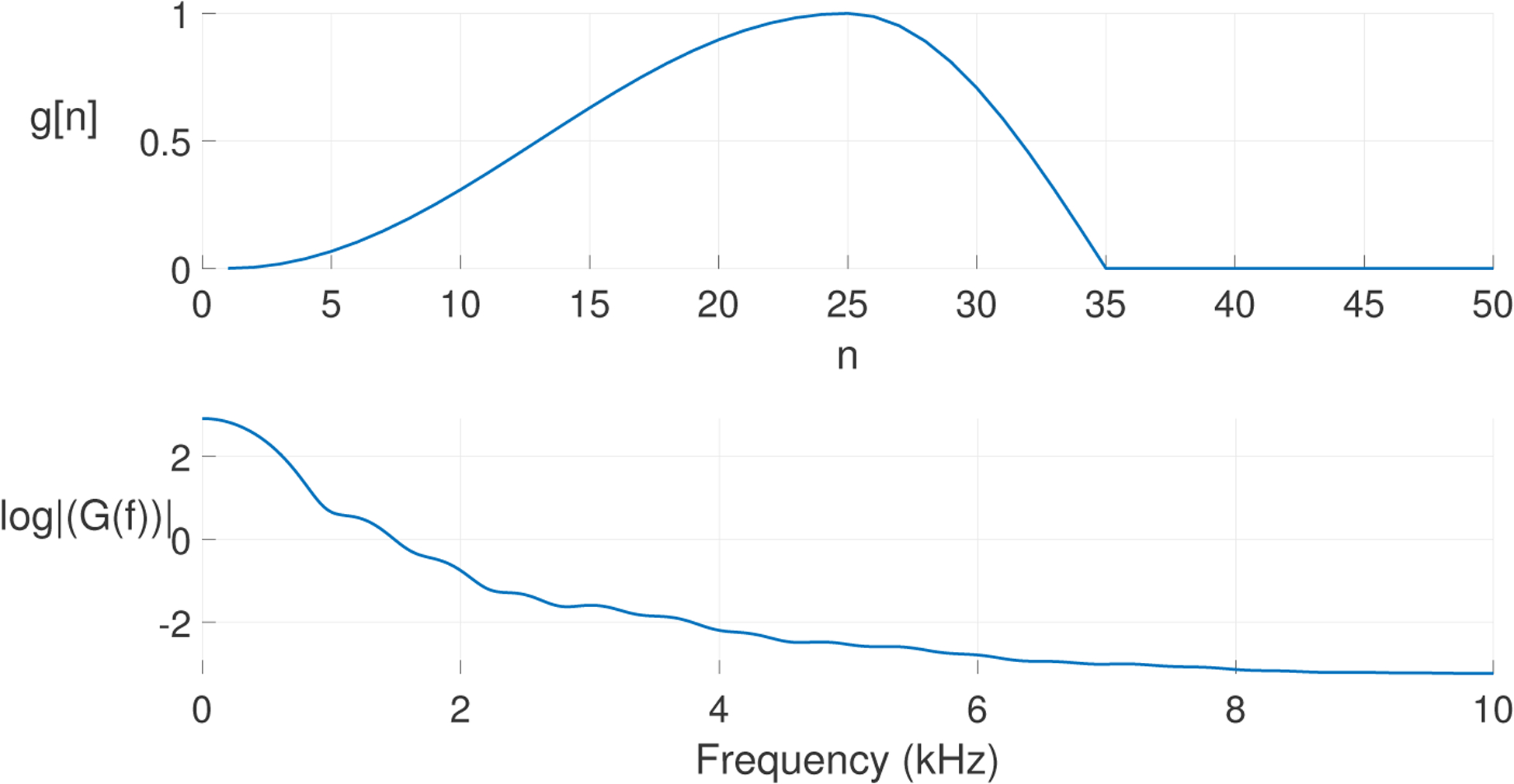
Rosenberg model in time domain (only first 50 samples shown, **top** panel) and the magnitude of its spectrum (**bottom** panel).

The glottal pulse time-domain waveform *g*[*n*] and its spectrum are plotted in Figure 1

The periodic comb excitation *p*[*n*] is modeled as one-sided quasi-periodic impulse train:

(14) $$p[n]=\sum_{k=0}^{\infty }\gamma^{k}\delta \left[n-kN_{p}\right],$$

which has *z*-transform:

(15) $$P(z)=\sum_{k=0}^{\infty }\gamma^{k}z^{-kN_{p}}=\frac{1}{1-\gamma z^{-N_{p}}},$$

where *N*_*p*_ = *f*_*s*_/*f*_0_ (fundamental period in samples) and *γ* is a number close to 1 (e.g., 0.999) to make the filter stable. The spectrum of the periodic input *P*(*z*) has a fundamental frequency of *f*_0_ = 210 Hz (*N*_*p*_ = 96).

**Figure 2.**
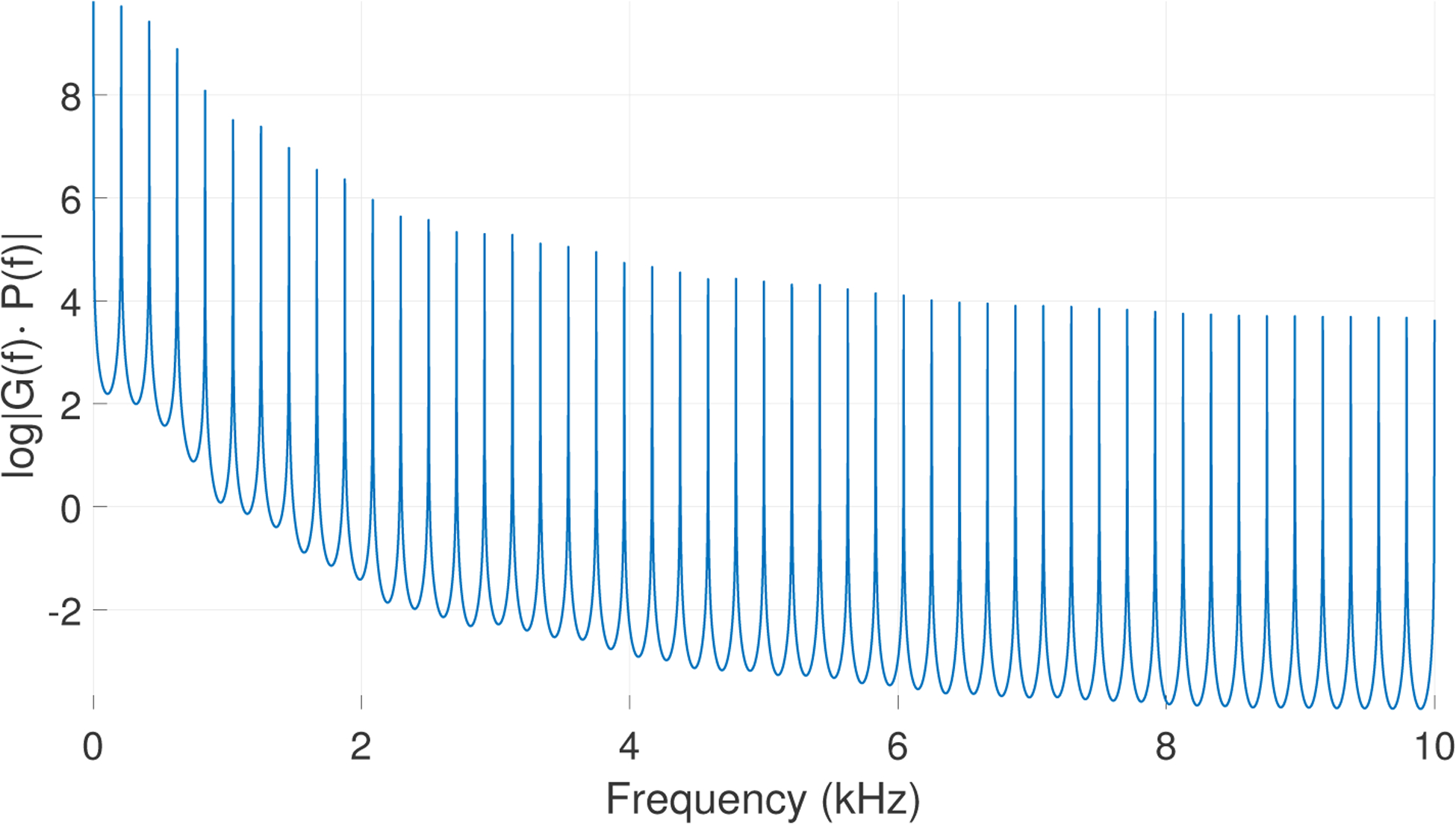
Spectrum of periodic input *P*(*z*) multiplied in the frequency domain by Rosenberg model *G*(*z*), which corresponds to an ARMA model of the glottal source (*f*_0_ = 210 Hz).

Therefore, *P*(*z*)*G*(*z*) is the *z*-transform of the glottal flow model (spectrum shown in Figure 2). In the time-domain, the GVV signal can be represented by an ARMA model that can be constructed as a shaping filter (*sf*) driving the canonical MA model (see Equation (16)) [43,44]:

(16) $$x_{sf}(n)=-\sum_{k=1}^{p}\alpha_{k}x_{sf}(n-k)+\sum_{k=0}^{q}\beta_{k}w_{2}(n-k),$$

where *x*_*sf*_ (*n*) is the state of the shaping filter, *α*_*k*_ = −*γ*_*k*_ and *β*_*k*_ are the *k*th coefficient of the AR and MA model, respectively, and *w*_2_(*n*) is Gaussian noise with mean 0 and variance $\sigma_{w_{2}}^{2}$ . The state-space equation for this model is:

(17) $$x_{SF}(n+1)=A_{SF}x_{SF}(n)+B_{SF}w_{2}(n),$$

(18) $$w_{1}(n)=C_{SF}x_{SF}(n),$$

where **x**_**sf**_(*n*) = (*x*_*SF*_(*n* − *p* + 1) *x*_*SF*_(*n* − *p* + 2) … *x*_*SF*_(*n*))^*T*^ is the state vector and *p* is the order of the AR model. Since the periodic input has *N*_*p*_ poles, the order of the AR model is *p* = *N*_*p*_. **A**_**SF**_ is the transition matrix *p* × *p*:

$$A_{SF}=\left[\begin{array}{cccccc} 0 & 1 & 0 & \ldots & 0 & 0\\ 0 & 0 & 1 & \ldots & 0 & 0\\ \vdots & \vdots & \ddots & \vdots & \vdots & \vdots \\ 0 & 0 & 0 & \cdots & 0 & 1\\ -\alpha_{p} & -\alpha_{p-1} & -\alpha_{p-2} & \cdots & -\alpha_{2} & -\alpha_{1} \end{array}\right]\in {\mathbb{R}}^{p\times p},$$

**Figure 3.**
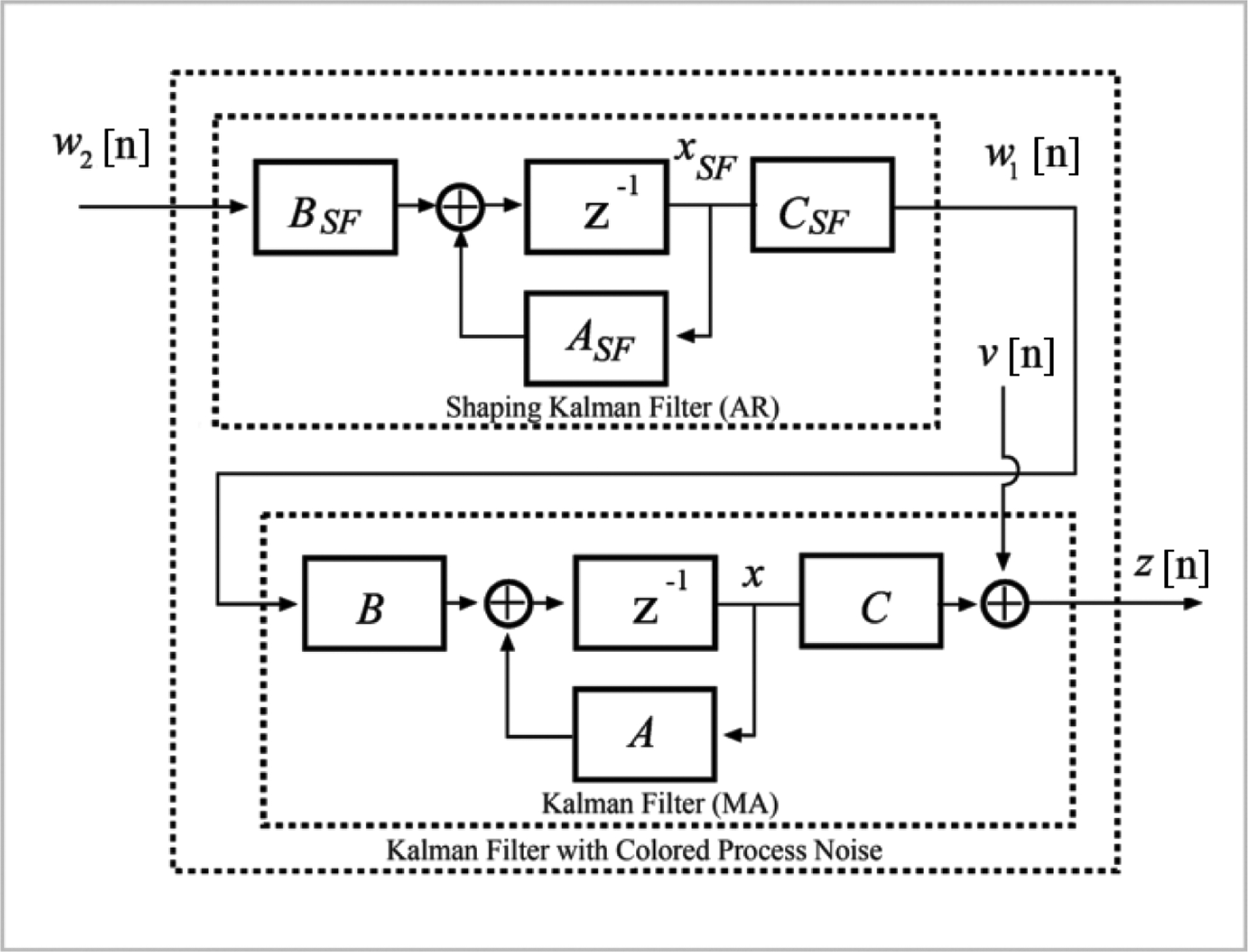
Diagram of modified Kalman Filter with colored state noise process. The physical system corresponds to the standard MA Kalman Filter, with a shaping Kalman filter based on a autoregressive noise process from the spectrum of a periodic Rosenberg glottal flow excitation.

$B_{SF}={\left[\begin{array}{c} \begin{array}{ll} 0 & 0\;\ldots \;1 \end{array} \end{array}\right]}^{T}\in {\mathbb{R}}^{p\times 1}$ and *w*_2_(*n*) is a stochastic driving noise with zero mean and variance $\sigma_{w_{2}}^{2}$. The MA equation Equation (18) contains $C_{SF}=\left[\begin{array}{lll} \beta_{q} & \beta_{q-2}\;\ldots \;\beta_{1} & \beta_{0} \end{array}\right]\in {\mathbb{R}}^{1\times (q+1)}$ and the colored noise $w_{1}(n)\in \mathbb{R}$ is the dot product of **C**_**SF**_ and **x**_**SF**_(*n*). Considering the source-filter theory, the colored noise model can be considered as modeling the GVV for the Kalman implementation of IBIF in Equation (19). A diagram of this augmented system is shown in Figure 3. The white noise *w*_2_(*n*) is the input to the shaping filter, the latter being the Rosenberg model convolved with the periodic input (Figure 2). The output of this filter is the colored noise *w*_1_(*n*) modeling the GVV signal, which is the state noise to the canonical MA system (physical system in Figure 3), whose output *z*(*n*) is the observed signal, i.e., the neck-skin acceleration. The new state-space equations in discrete-time are:

(19) $$X_{T}(n+1)=A_{T}X_{T}(n)+B_{T}w_{2},$$

(20) $$z(n)=C_{T}X_{T}(n)+v(n),$$

where

(21) $$A_{T}=\left[\begin{array}{cc} A & C_{SF}\\ 0 & A_{SF} \end{array}\right]$$

(22) $$B_{T}=\left[\begin{array}{c} 0\\ B_{SF} \end{array}\right]$$

(23) $$C_{T}=\left[\begin{array}{c} \begin{array}{ll} C & 0 \end{array} \end{array}\right]$$

(24) $$X_{T}=\left[\begin{array}{c} x\\ x_{SF} \end{array}\right].$$

**Figure 4.**
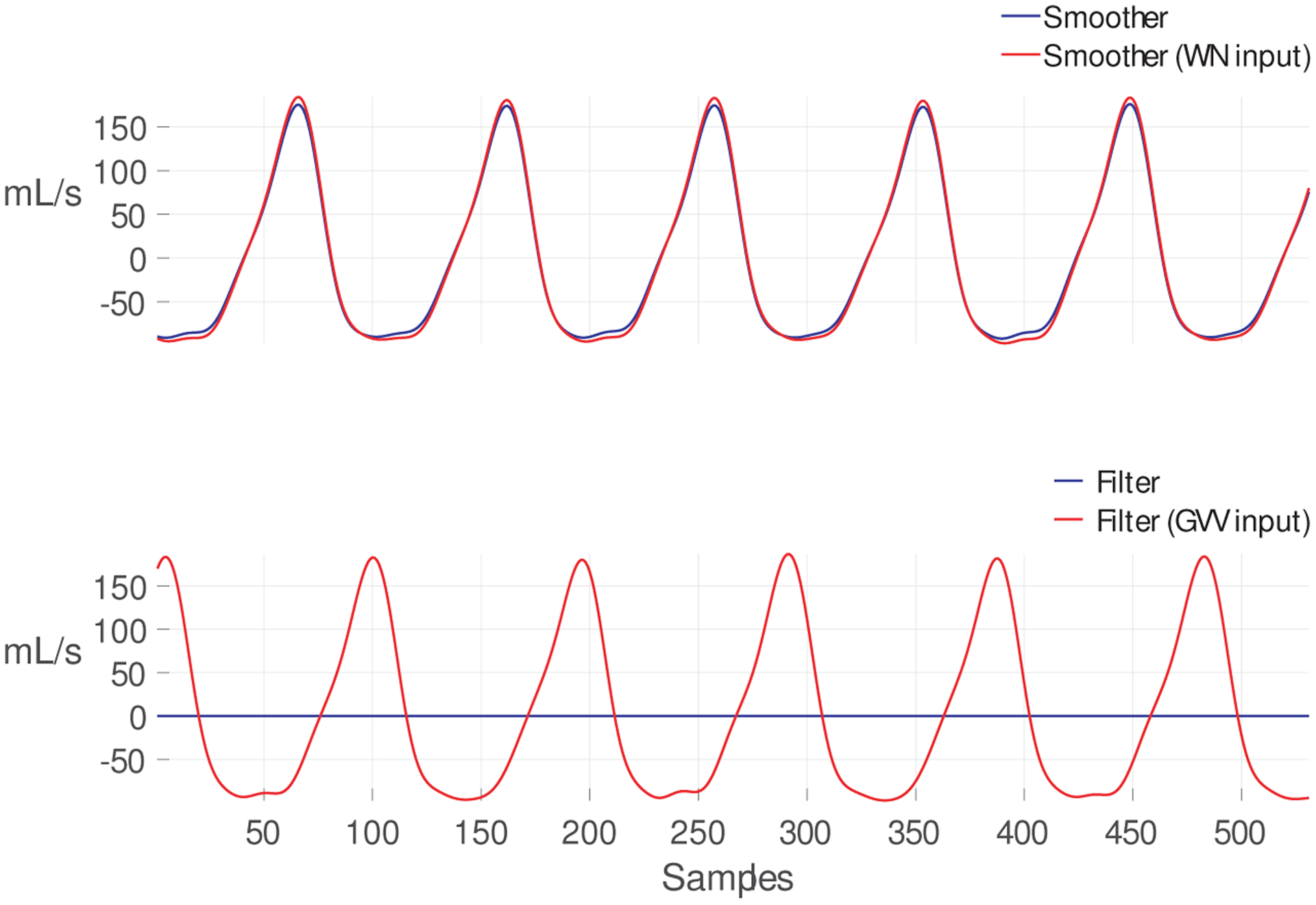
**Top** panel: GVV estimates $\left(\hat{x}(n-N+1|n)\right)$ using **A** (blue) and **A**_**lp**_ (red). **Bottom** panel: GVV estimates $\left(\hat{x}(n|n)\right)$ using **A** (blue) and **A**_**lp**_ (red).

An example of the estimated GVV using matrix **A**_**T**_ is shown in Figure 4 and compared to the estimated GVV using the original matrix **A**. The upper plot shows the tracking of the first time step state $\hat{x}(n-N+1|n)$, which corresponds to the smoothed (time delayed estimate) GVV. Can be noticed that there are no differences between the original MA Kalman filter and the one incorporating a colored state noise. There is, however, a noticeable difference in the tracking of the last time step state $\hat{x}(n|n)$ of the GVV, which corresponds to the the filtered GVV estimate considering all the observation information up to the current sample *n*. The original Kalman filter produces a zero-mean signal, while the modified Kalman filter with colored state noise modeling the glottal spectrum tracks an expected GVV signal.

**Figure 5.**
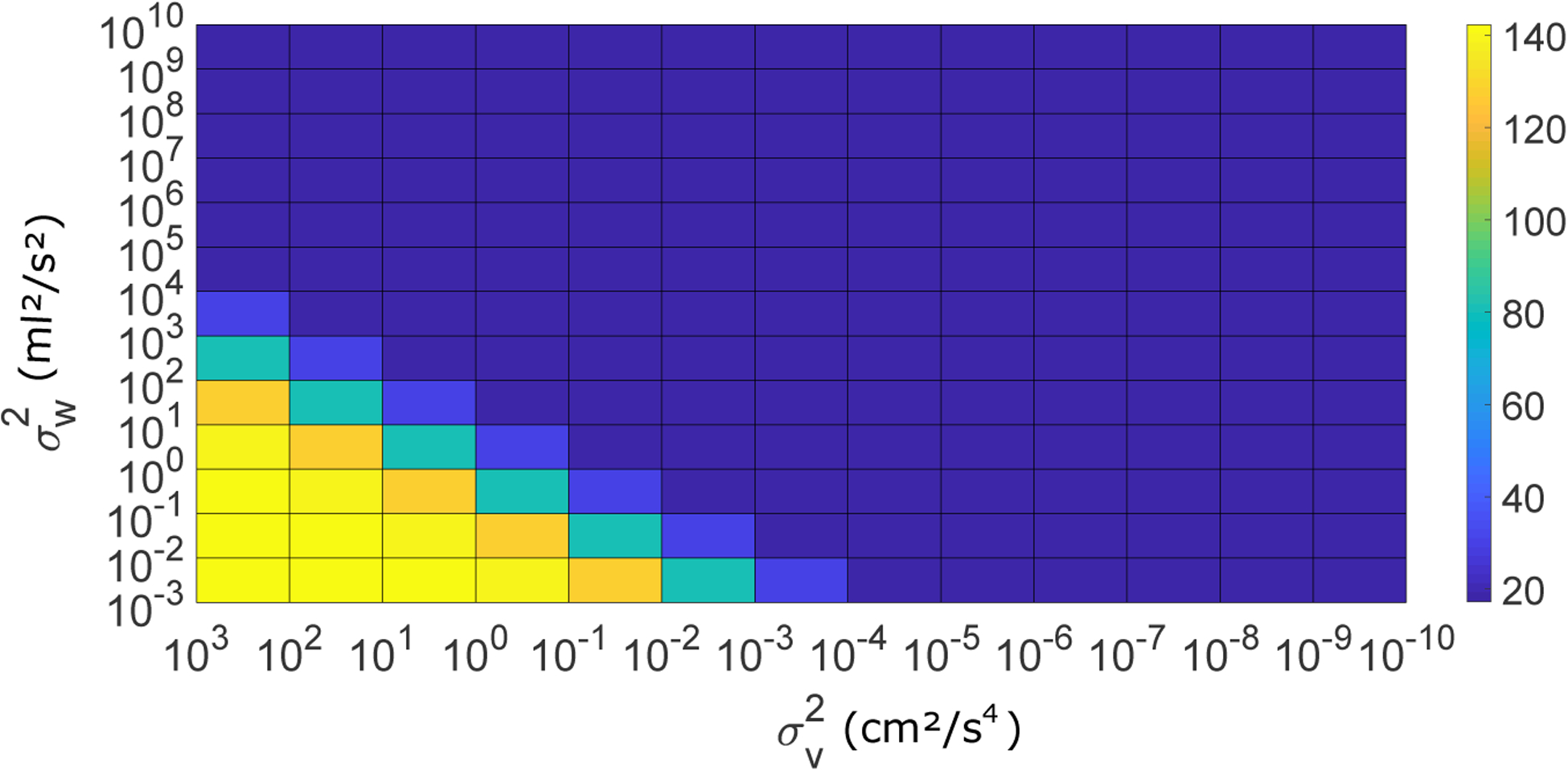
RMSE values for different combinations of $\sigma_{w}^{2}$ and $\sigma_{v}^{2}$.

The proposed implementation of the IBIF method in a Kalman filter framework has two important additions: the adaptive tracking of the GVV signal using the accelerometer and the modeling of state and observation noise. In the first case, the adaptive tracking is performed through the sample by sample correction of the predicted accelerometer signal by the Kalman gain **K**(*n*). In our hypothesis, the correction term helps to improve the estimation of the GVV signal by minimizing the deviations from the GVV signal obtained with IBIF. The process noise variance $\sigma_{w}^{2}\left({\text{mL}}^{2}/{\text{s}}^{2}\right)$ (mL^2^/s^2^) and the observation noise variance $R_{v}=\sigma_{v}^{2}\left({\text{cm}}^{2}/{\text{s}}^{4}\right)$ (cm^2^/s^4^) were selected using a grid-search process to compare the root-meansquare error (RMSE, mL/s) between the Kalman state *x*(*n* − *N* + 1) and a reference GVV signal obtained by inverse filtering of the OVV signal [9]. Figure 5 shows different values of $\sigma_{w}^{2}$ and $\sigma_{v}^{2}$ where multiple minima (*RMSE* = 17.268) are found within a range for one subject producing the vowel /a/. Most blue RMSE values in Figure 5 correspond to *RMSE* = 17.273 which are very close to the minimum. Similar trends were found for other subjects and vowels. We selected $\sigma_{w}^{2}=100$ and $\sigma_{v}^{2}=1$ in this work, which are plausible values for the state and measurement noises due to the assumption of higher process noise due to glottal flow variance with low observation noise, while they produce the minimum RMSE value.

## 3. Experimental Setup

The human studies protocol used to collect the data for this study was approved by the Institutional Review of the Mass General Brigham (formerly, Partners Healthcare System) at the Massachusetts General Hospital. Study participants were 50 pairs of adult females (total of 100 subjects) with each pair comprised of one patient with PVH (diagnosed with vocal fold nodules) and one normal control subject matched to the patient by age and occupation. Due to the higher incidence of female patients with PVH than male in the overall population [52,53] and potential sex-specific effects (e.g., due to differences in fundamental frequency), only females were selected for this study. The patient matching was done to normalize for general vocal behavior differences. Clinical diagnoses were based on a complete team evaluation by laryngologists and speech-language pathologists at the Massachusetts General Hospital Voice Center that included (a) a complete case history, (b) endoscopic imaging of the larynx, (c) aerodynamic and acoustic assessment of vocal function [54], (d) a patient-reported Voice-Related Quality of Life questionnaire [55], and (e) a clinician-administered Consensus Auditory-Perceptual Evaluation of Voice assessment [56]. All patients were enrolled prior to the administration of any voice treatment. Written informed consent was obtained from all subjects. The average (standard deviation) age of all subjects was 25.0 (10.5) years old.

**Figure 6.**
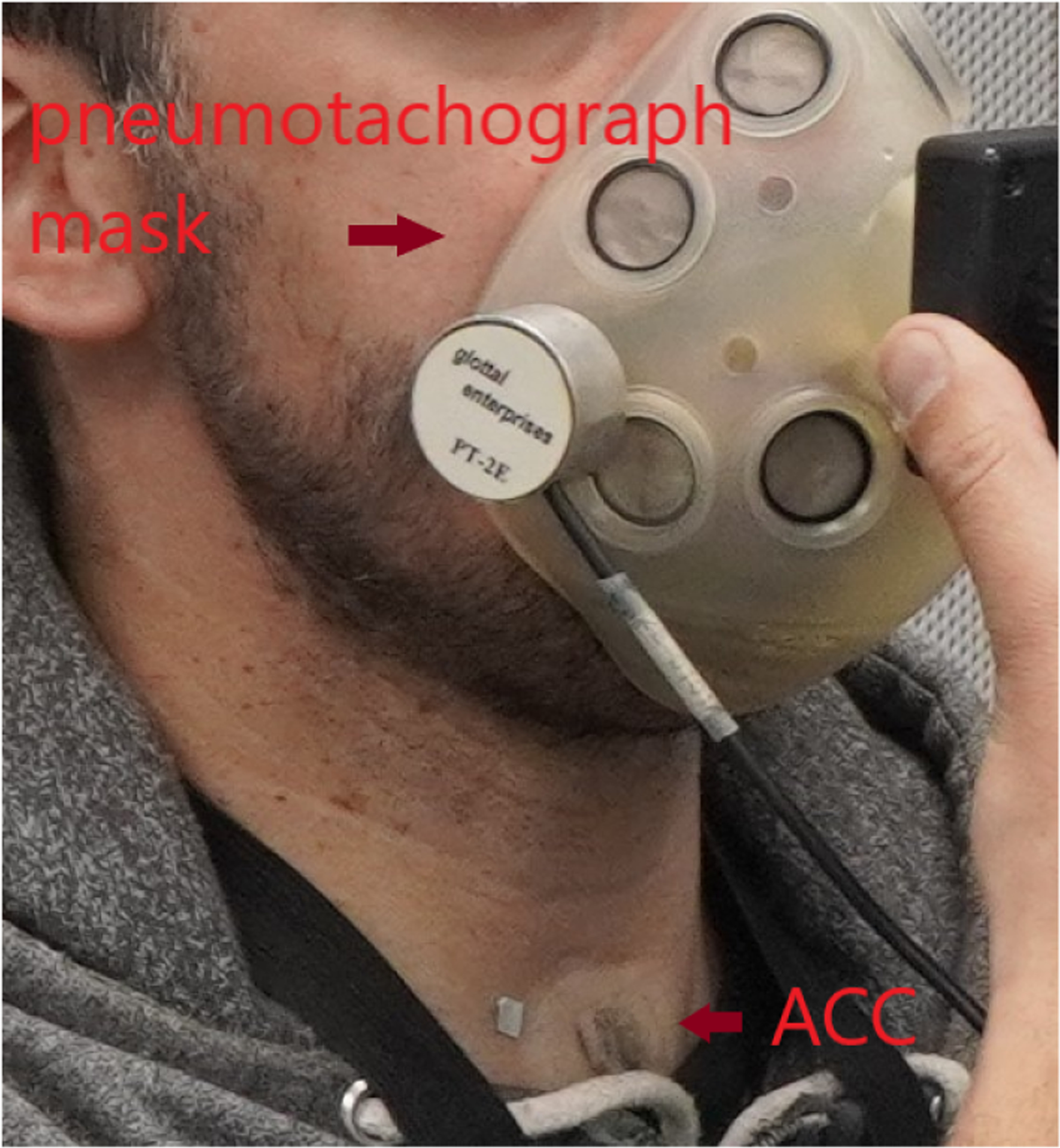
Experimental setup with oral airflow (pneumotachograph) mask and accelerometer sensor (ACC) on neck-surface location.

Each subject was recorded reading a phonetically balanced text (Rainbow Passage, [57]), at a comfortable loudness level, using a Voice Health Monitor system that consists of an accelerometer attached to the front of the neck below the larynx and connected to an smartphone application [14]. Also, synchronized recordings of oral airflow volume velocity provided a reference signal from which glottal airflow could be extracted using standard inverse filtering [32]. The sampling frequency for each signal is 20,000 Hz with an average of 30 s per passage. A typical set-up of the accelerometer attached to the neck surface is shown in Figure 6.

### 3.1. IBIF Calibration

Each subject underwent a session in the laboratory to obtain a subject-specific calibration for the IBIF algorithm. The session involved simultaneous and synchronous recordings of a circumferentially vented mask-based OVV and neck-surface acceleration in an acoustically treated room. Each subject performed a series of sustained vowel gestures (/a/ and /i/) with a constant pitch using comfortable and loud (approximately 6 dB increase) voice. For each gesture, a bandpass-filtered (60–1100 Hz) oral airflow vowel segment was used to perform inverse filtering with a single notch filter (SNF) constrained to unitary gain at DC [35,36].

Once a glottal airflow approximation was obtained from the OVV signal, the previously introduced ***Q*** parameters were estimated using the optimization scheme described in [33]. These are the parameters describing the mechanical properties of the neck skin, as well as the length of the trachea and the position of the accelerometer with respect to the glottis [33].

### 3.2. Ground Truth GVV

A ground truth GVV signal is necessary to compare the performance of the proposed algorithm. However, a measurement of GVV is infeasible because there is no sensor available to directly measure the airflow in the glottis. An alternative is to obtain a GVV estimate from an external sensor, e.g., an oral flow mask. Following the same method for IBIF calibration (Section 3.1), the SNF method is used in this work to calculate the ground truth GVV. Even though this ground truth is an estimation of the true glottal flow (due to the difficulty of obtaining directly the latter signal), the SNF method has been successfully applied in previous work related to GVV estimation in sustained vowels [5,9,29,36,58]. Since we have running speech in this case, the optimization procedure that finds the best notch frequency and bandwidth is done in every 50 ms non-overlapping frame. A simple voice activity detector based on the autocorrelation method [51] is used to remove unvoiced frames. The signal is reconstructed from individual frames by using the overlap-and-add method [51].

### 3.3. Reducing Order of the IBIF Filter

**Figure 7.**
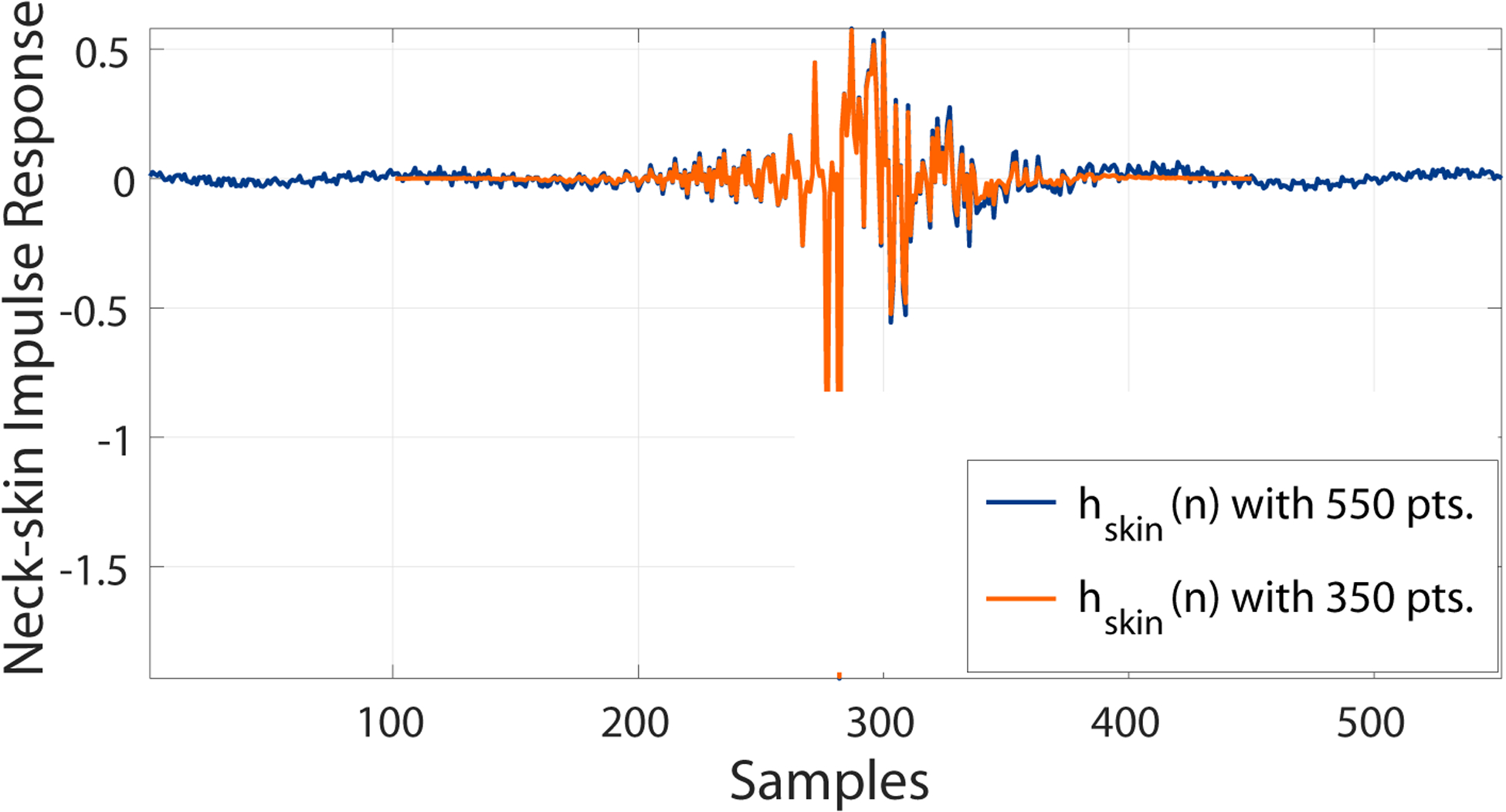
Neck-skin impulse response for a healthy female subject, full impulse (blue) and truncated version with a Hanning window (orange).

**Figure 8.**
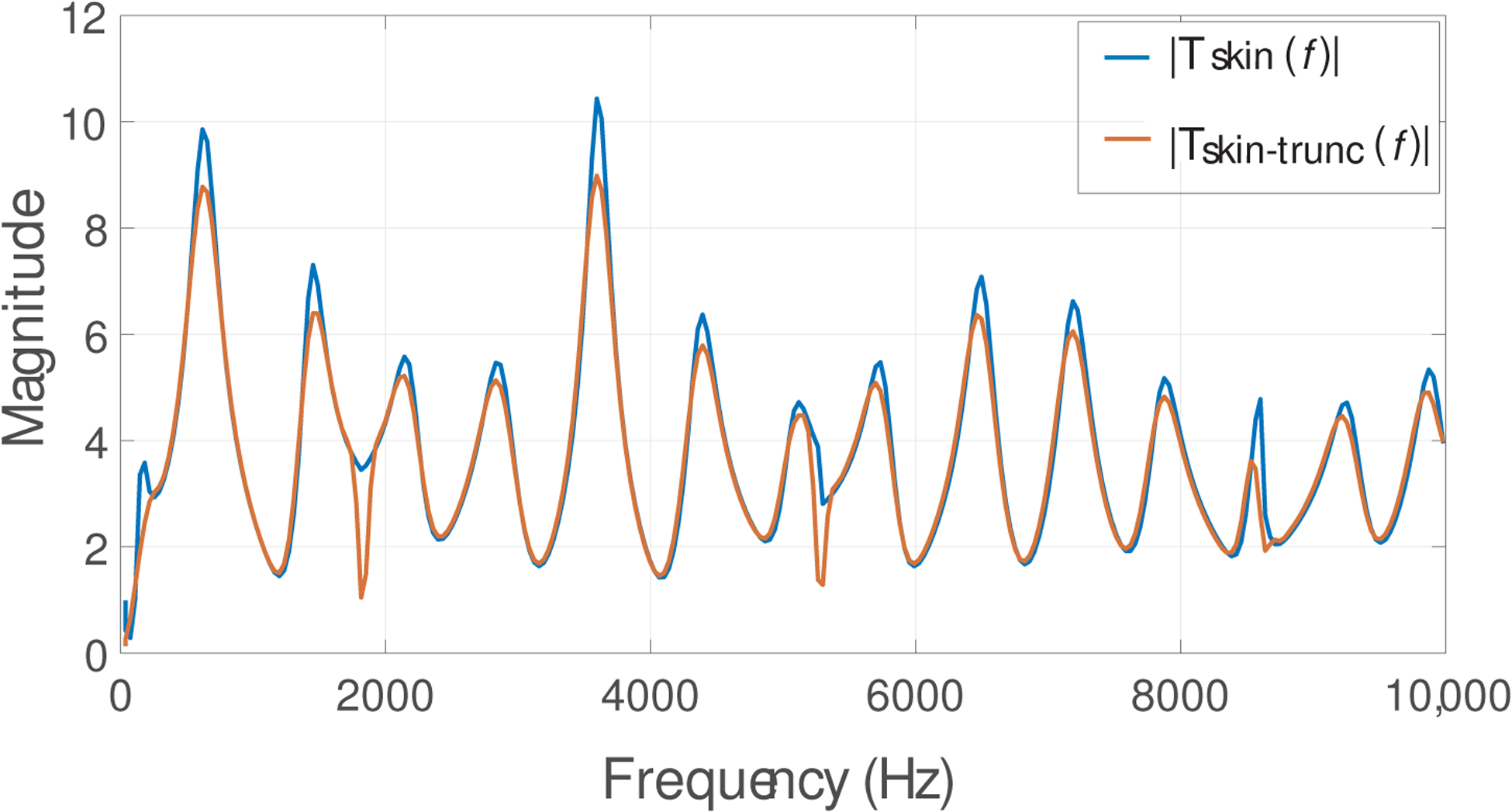
Neck-skin frequency response for a healthy female subject, full length (blue) and truncated version with a Hann window (red).

In order to reduce the complexity of the Kalman filter, we need to reduce the size of the matrices **A** and **C** in Equations (10) and (11). This is necessary due to the computational cost of Kalman filter in the multiplications of state-space matrices of size 550 × 550 when processing running speech. Since **A** and **C** depend on the length of the neck-skin impulse response *h*(*n*), the latter is truncated in the middle region and then windowed (Hanning function) to 350 points. This procedure seeks to maintain the performance of IBIF filter because most of the energy of the impulse response is concentrated in the middle section, while the extremes are considerably low in energy. As an example, Figure 7 shows a given *h*(*n*) in blue and the resulting truncated version in orange. The magnitude of the frequency response is shown in Figure 8.

### 3.4. Aerodynamic Features

**Figure 9.**
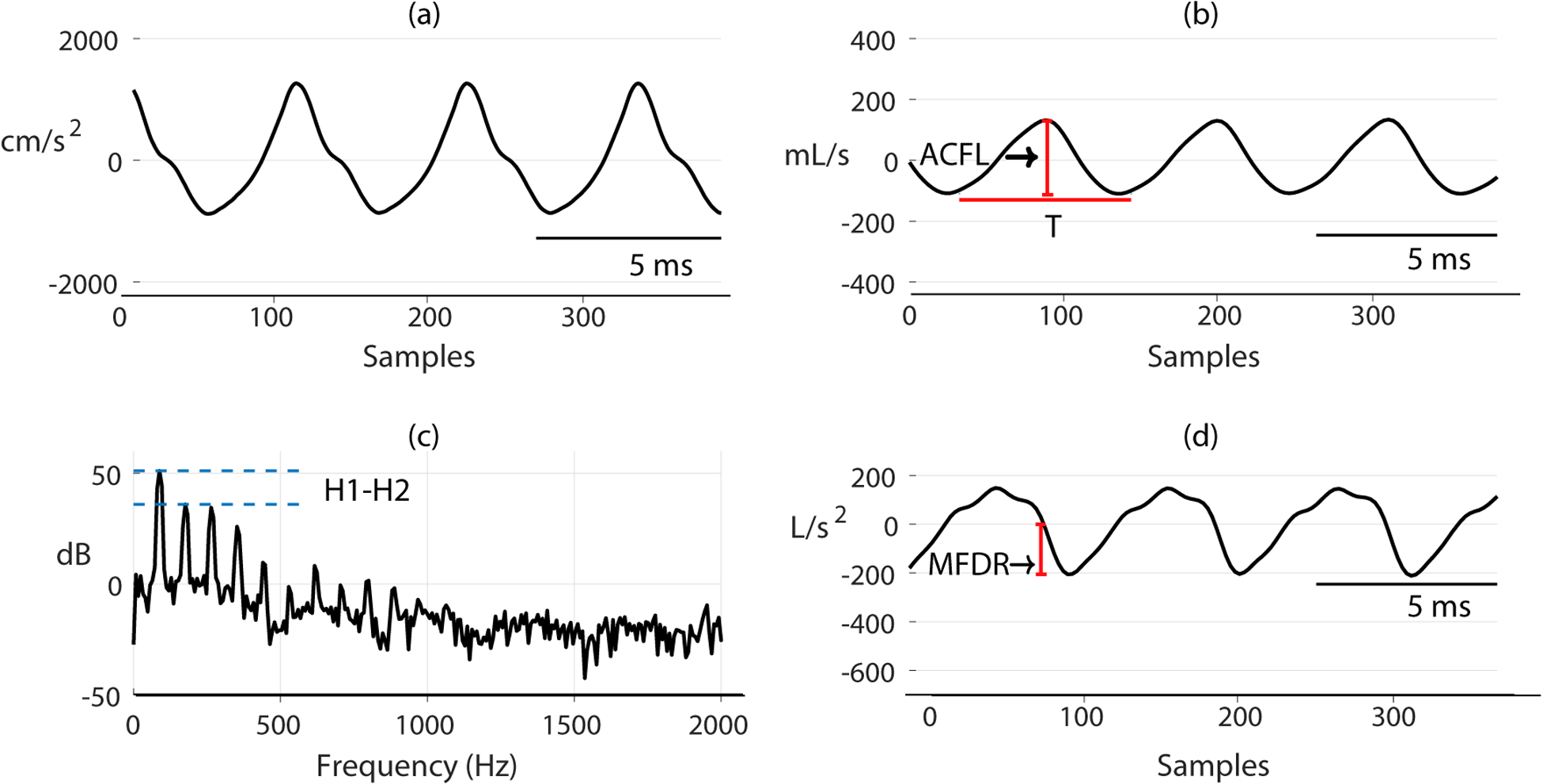
(**a**) ACC frame, (**b**) GVV frame, (**c**) spectrum from (**b**), and (**d**) time-derivative from (**b**).

The GVV signals from IBIF, SNF, and Kalman methods are divided in 50 ms, non-overlapping frames. Voicing is detected by calculating the normalized autocorrelation of the ACC signal and the main peak exceeding a threshold of 0.8. If the frame is voiced, measures are extracted from the GVV waveform, its time-derivative, and spectrum. Figure 9a shows an ACC frame and (b) a GVV waveform, the spectrum (c) and the time-derivative waveform (d). The features used in this work are described in Table 1. Some of these aerodynamic features, such as AC flow (ACFL) and maximum flow declination rate (MFDR), have been shown to be useful to discriminate between subjects with PVH and healthy controls [5,9,16,29,58]. Instead of estimating time-domain features based on the detection of glottal opening and/or closing instants, the normalized amplitude quotient (NAQ) is calculated in this study, due to its robustness to noisy measurements and its correlation to the close quotient of the glottal cycle [59]. For time-domain measures (ACFL, MFDR, NAQ and *f*_0_), the median for all cycles within the 50 ms frame is obtained. The difference in magnitude of the first and second harmonic (H1–H2) is computed from the GVV spectrum.

## 4. Results

**Figure 10.**
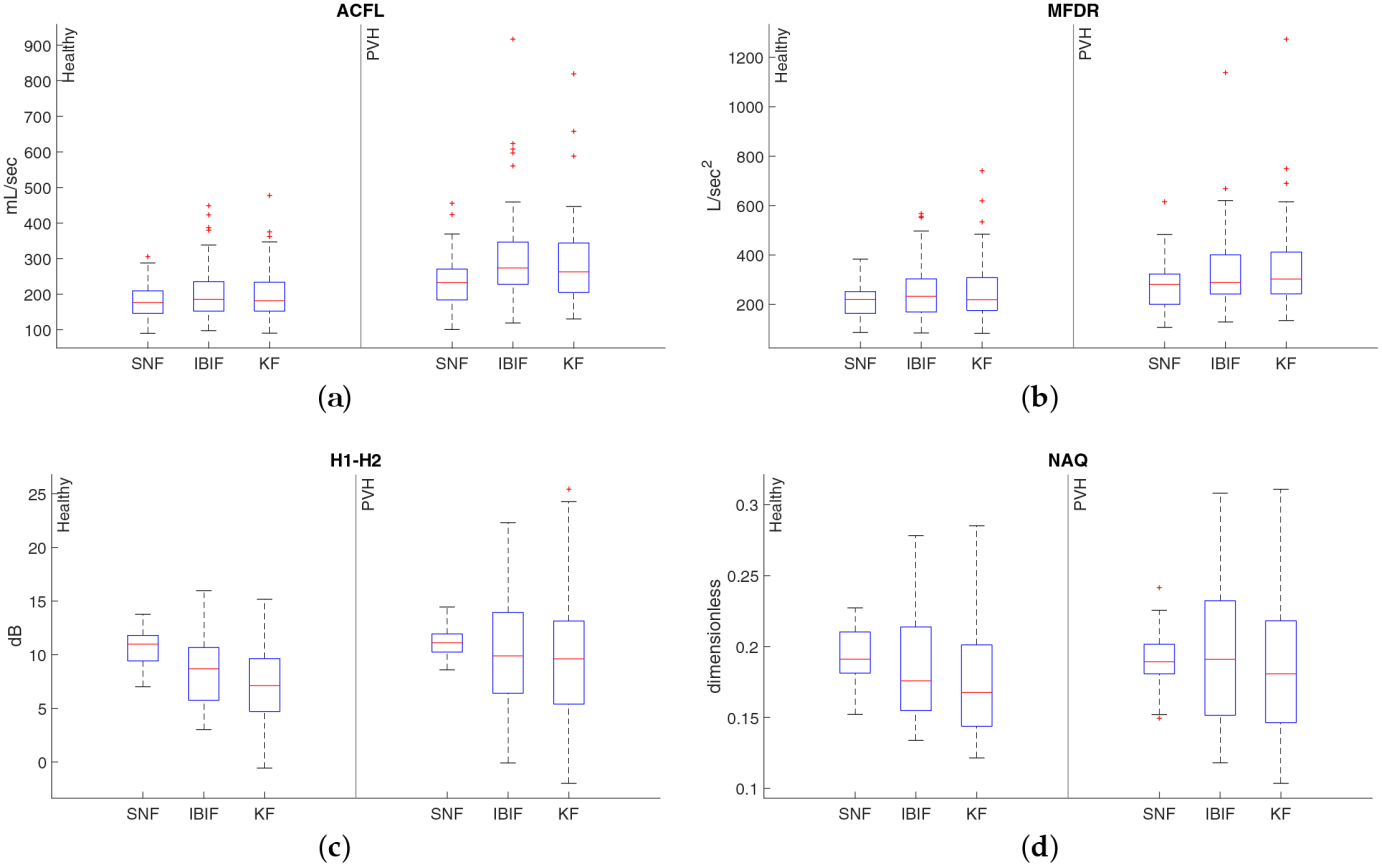
Distribution of average measures from the Rainbow passage for 50 vocally-healthy subjects (left panel in each subplot) and 50 PVH subjects (right panel in each subplot): (**a**) ACFL, (**b**) MFDR, (**c**) H1–H2, and (**d**) NAQ

Table 2 shows summary statistics (mean ± standard deviation) of average values, per subject, of ACFL, MFDR, H1–H2, NAQ, and *f*_0_ from the Rainbow passage speech data, across PVH and healthy subjects, calculated with SNF, IBIF, and KF implementation of IBIF with colored noise model. Mean values are not statistically different for the three methods. Figure 10 shows box plots for some of the measures. Overall, the distribution of measures is similar when using the standard IBIF and the modified Kalman filter algorithm.

From Table 3, the mean values of ACFL and MFDR from healthy subjects are not significantly different between the standard IBIF, the modified Kalman filter, and the ground-truth GVV (one-way ANOVA: *F* = 1.8, *p* = 0.2 for ACFL, *F* = 2.7, *p* = 0.07 for MFDR). Therefore, both IBIF and the modified Kalman filter have similar ACFL values comparable to the ground-truth GVV. Instead, ACFL from PVH subjects are significantly different between the standard IBIF and the ground-truth GVV (*F* = 4.1, *p* = 0.02), while the modified Kalman does not have significant differences withe the same ground-truth. Similar to ACFL from healthy subjects, MFDR from the same group do not show significantly differences between the two methods and the ground-truth GVV. However, there was a small but significantly difference between the modified Kalman filter and the ground-truth GVV for PVH subjects (*F* = 3.3, *p* = 0.04), indicating that MFDR from the modified KF does not estimate MFDR as well as the standard IBIF, when compared to the ground-truth of that group. For all other measures, there were not significantly differences, in which either case, the modified KF or standard IBIF could provide similar mean results comparable to the ground-truth measure.

The root-mean-square-error (RMSE) between the KF implementation and the ground-truth GVV (*RMSE*_*KF*_) and the RMSE between the standard IBIF and the ground-truth GVV (*RMSE*_*IBIF*_) were calculated for each subject with voiced frames from the Rainbow Passage. The percentage of the error difference Δ = (*RMSE*_*KF*_ − *RMSE*_*IBIF*_)/*RMSE*_*IBIF*_ are shown in Table 4 as the median and interquartile range for each PVH and healthy group for all the glottal features. The results indicate an improvement on the median of ACFL for both healthy and pathological using the KF implementation compared to the standard IBIF. Other features show medians indicating IBIF provides a better estimate of the ground-truth signal. However, it is worth to notice that there is a large dispersion of Δ’s for all subjects, indicating that some subjects estimates have a large improvement by using KF as well. Moreover, ACFL estimated from the neck-surface acceleration signal is a key measure able to discriminate between PVH subjects from healthy controls [29] in steady vowels, and which the KF implementation can provide better estimates.

**Figure 11.**
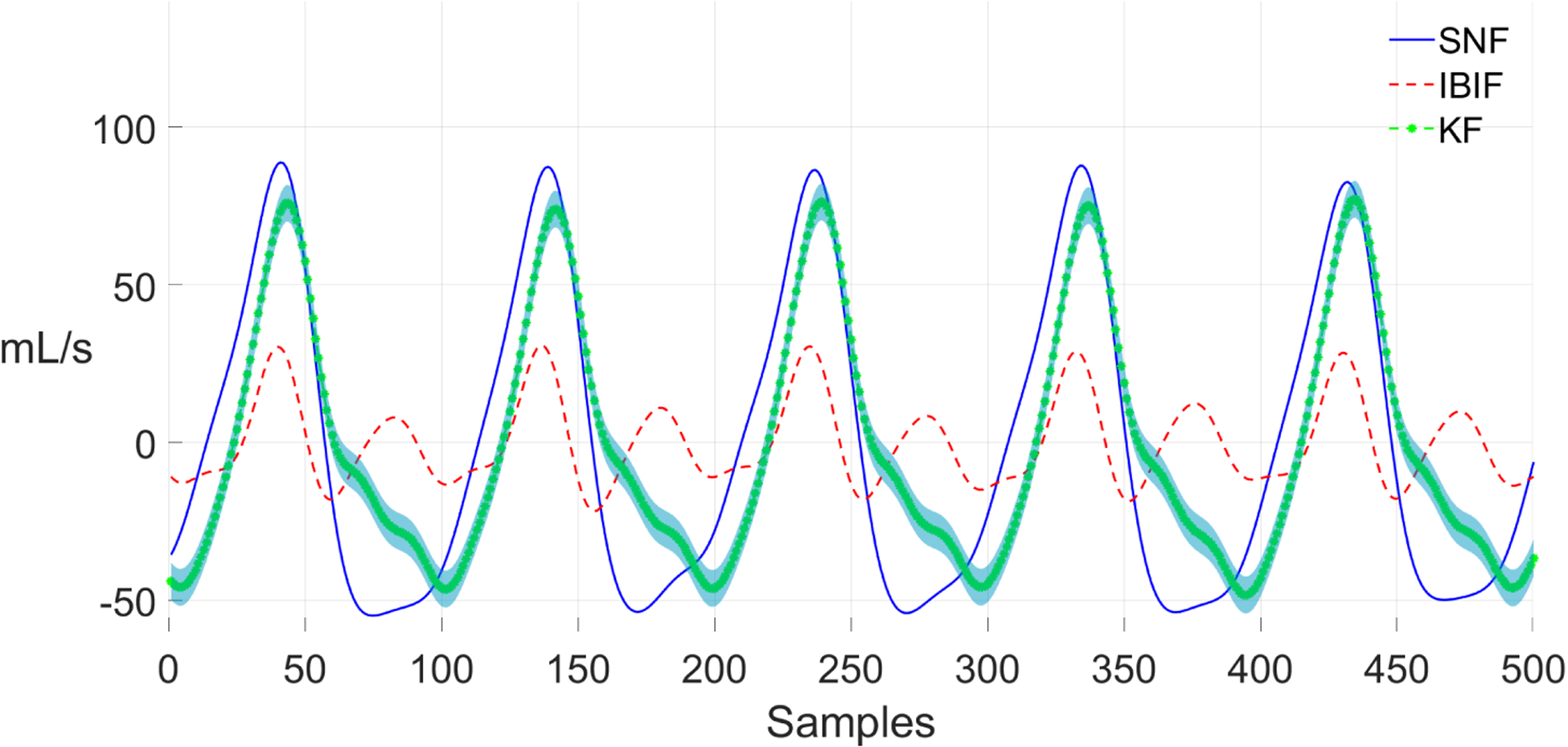
Section from the Rainbow passage (healthy female) with estimations of GVV: Kalman filter (solid and dot green), IBIF (dashed red), and single notch filter (solid blue). The estimation of GVV using Kalman filter includes ±2*σ* (standard deviation) on the green shaded region.

We can observe some differences between the IBIF and its KF implementation when estimating the peak-to-peak amplitude (e.g., ACFL). Figure 11 shows a voiced segment of the Rainbow passage from a vocally healthy female. The KF method (green line) estimates a reasonably good fit to the GVV waveform from the SNF method (*RMSE* = 24.9 mL/s). However, the IBIF method does not follow the same ground truth signal (*RMSE* = 42.7 mL/s). The peak-to-peak amplitude is smaller, and the close phase contains a large resonance. However, the KF method improves the estimation of the peak-to-peak amplitude for the same segment. There is some phase distortion in the closed and opening phase, but overall, the waveform has a closer match to the SNF method than IBIF. The errors to IBIF could be attributed to the production of vowels whose spectra are substantially different to a steady /a/ vowel, which in some cases could affect estimated glottal features up to 50% in error [60].

**Figure 12.**
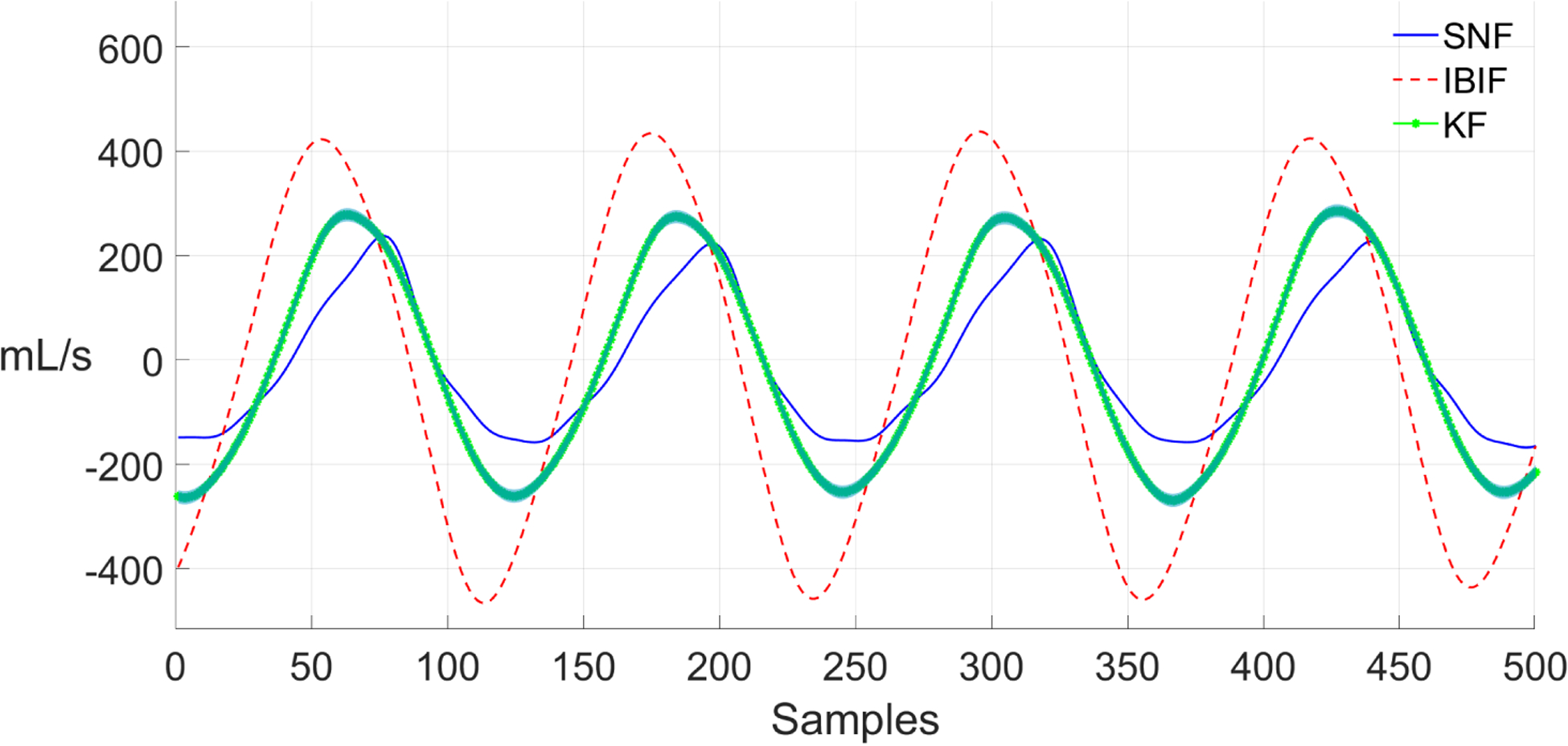
Section from the Rainbow passage (female PVH) with estimations of GVV: Kalman filter (solid and dot green), IBIF (dashed red), and single notch filter (solid blue). The estimation of GVV using Kalman filter includes ±2*σ* (standard deviation) on the green shaded region.

Figure 12 shows a voiced segment from a PVH female subject. In this case, the IBIF methods overestimates the peak-to-peak values from the SNF method (*RMSE* = 229.4 mL/s). Also, the opening phase is faster compared to ground truth. The KF method compensates the large amplitude of the IBIF output waveform, while at the same time it gets closer to the ground truth signal in the opening and close phase (*RMSE* = 76.3 mL/s). As previously stated, the IBIF and, therefore, the model used by the Kalman filter, are both calibrated using a procedure based on fitting the vowel /a/. In these cases, the method based on Kalman follows the reference signal a bit closer than IBIF. Even though the Kalman filter is an alternative implementation of the IBIF filter, the adaptive filtering nature of Kalman allows to track better the ground truth signal than IBIF. Similar trends were found in different subjects and tokens.

## 5. Discussion

The proposed method based on the modified MA framework and the Kalman filter algorithm is an adaptive implementation of the IBIF scheme. Therefore, it has some differences with the original IBIF design, namely a forward prediction of the accelerometer signal (i.e., no filter is inverted) and a truncation of the finite impulse response required to reduce the computational burden. Despite these differences, in this paper we have shown that the Kalman filter implementation allows for enhancing the glottal airflow estimates, as it optimally adapts its latent states to better predict the accelerometer signal, thus resulting in a closer estimation of the glottal airflow from a Rothenberg mask in benchmark experiments. It is important to note that there are still differences between the Kalman filter glottal airflow estimates and the reference signal from the Rothenberg mask, due to supraglottal inverse filtering errors and measurement uncertainty of the oral airflow signal [61]. Small, but significant, differences between the mean values of ACFL and MFDR from PVH subjects can be observed using the IBIF and Kalman method, respectively. These are difficult to assess, particularly, for high-pitched female pathological voices [62]. For example, the method of closed phase covariance requires several samples in the closed phase of the glottal flow, which are difficult to obtain for high-pitched subjects [63].

The signal deviations between the Kalman filter and the original (time invariant) FIR IBIF glottal airflow estimates are relatively small, although the former better estimates the amplitude, or peak-to-peak flow, compared to IBIF. These differences can be relevant in some cases, depending on the application. In the case of ACFL, there is an improvement on its estimation using KF from running speech, which adds value in a clinical setting, where ACFL has proven to be a key discriminant measure between health subjects and subjects with PVH only for steady vowels [29]. When assessing the relevance of these differences in the context of a classification task to discriminate between vocal fold nodules patients and control subjects using ambulatory accelerometer data, no significant variations in the classification were found, even when comparing frames with low and high error (or deviation) [64]. Thus, the classification task for long periods of time seems to be fairly insensitive to the uncertainty of the airflow estimates from IBIF model parameters, sensor positioning, and other effects. This supports the use of the original FIR version of the IBIF scheme for such classification tasks, which indicates that factors affecting the classification performance in [16] were not degraded by the airflow estimates. However, other applications more sensitive to signal quality (for instance, the estimation of glottal biomechanics and assessment of tissue-flow-acoustic interaction [65]) can further benefit from the enhancement offered by the proposed Kalman implementation to estimate more accurate glottal airflow in running speech and/or ambulatory scenarios.

The main differences between the Kalman filter and SNF approach can be observed in H1–H2 and NAQ measures, which are related to low-frequency content and closed quotient, respectively. The IBIF method shows similar differences as well. In order to better estimate these measures, it is necessary to correctly detect the upward and downward slope of the glottal cycle, as well as the closed phase portion. Undue modelled rapid changes in the signal trajectory might induce errors in the Kalman approach which affect the detection of those landmarks in the glottal cycle. In addition, for some subjects, errors in the parameters from IBIF due to calibration could carry through to the Kalman implementation since the latter is built upon the IBIF scheme.

The main current limitation of the proposed Kalman filter approach is the relatively high computational cost due to the FIR model used, which can become a problem when processing many hours of recordings (as in ambulatory monitoring) in numerous subjects. Future efforts can be devoted to optimizing the approach via more efficient methods, using for example an autoregressive model in the construction of the state space model. Also, an optimal tuning of process and observation covariance matrix can be explored to improve the estimation. Other variations in the construction, e.g., addition of a random walk term or an extended Kalman filter could be investigated as well to encompass non-linear implementations of the accelerometer signal to glottal airflow signal transformation. Flow estimation can also be improved by considering the backward Kalman smoother algorithm, at the expense of an increase in the computational burden and the memory requirements. New model strategies suitable for Kalman filter and smoother would be explored in the future [66].

## 6. Conclusions

A Kalman filter implementation of the subglottal impedance-based inverse filtering scheme was introduced to enhance the estimated glottal airflow from recordings of a neck-surface vibration signal and to assess the relevance of model uncertainty in such estimates. The proposed approach can adapt the signal estimates to correct for inverse filtering deviations, as observed in benchmark experiments with different sustained vowels. Future work is related to the exploration of other applications that can further benefit from the Kalman filter enhancement when estimating glottal airflow and to reduce its computational complexity.

## Figures and Tables

**Table 1.** Frame-based derived glottal airflow measures.

Glottal Airflow Measures	Description	Units
ACFL	Peak-to-peak glottal airflow	mL/s
MFDR	Negative peak of the first derivative of the glottal waveform	L/s^2^
H1–H2	Difference between the magnitude of the first two harmonics	dB
Normalized Amplitude Quotient (NAQ)	Ratio of ACFL to MFDR divided by the glottal period	–
Fundamental frequency (*f*_0_)	Inverse of the glottal period	Hz

**Table 2.** Mean and ± standard deviation from a pool of average values of ACFL, MFDR, H1–H2, NAQ, and *f*_0_ extracted from the Rainbow Passage (voiced frames only).

		ACFL	MFDR	H1–H2	NAQ	*f*_*0*_
PVH	SNF	238.8 ± 74.9	279.5 ± 102.0	11.1 ± 1.30	0.19 ± 0.02	202.4 ± 20.1
	IBIF	306.8 ± 147.7	346.8 ± 178.6	10.3 ± 4.89	0.20 ± 0.05	202.6 ± 20.3
	Kalman	287.6 ± 131.3	357.3 ± 199.1	9.69 ± 5.51	0.19 ± 0.05	200.8 ± 20.1
Healthy	SNF	184.5 ± 47.0	199.7 ± 77.8	10.7 ± 1.49	0.19 ± 0.02	204.6 ± 20.9
	IBIF	212.2 ± 82.9	260.0 ± 122.1	8.73 ± 3.63	0.19 ± 0.04	204.6 ± 21.0
	Kalman	199.7 ± 77.8	266.1 ± 138.3	7.73 ± 3.85	0.18 ± 0.04	203.5 ± 21.7

**Table 3.** One-way ANOVA table with mean values of glottal flow features for both Healthy and PVH subjects when comparing the standard IBIF, the modified Kalman filter, and the ground-truth GVV (* Statistically differences: *p* < 0.05).

	ANOVA	ACFL	MFDR	H1–H2	NAQ
Healthy	F	1.79	2.69	10.9	2.36
	*p*-value	0.17	0.07	* >0.001	0.1
PVH	F	4.12	3.27	1.39	0.74
	*p*-value	*0.02	*0.04	0.25	0.48

**Table 4.** Median (interquartile range) of RMSE Δs in percentage (%) of the modified Kalman filter with respect to the standard IBIF.

	ACFL	MFDR	H1–H2	NAQ
Healthy	−9.28 (41.6)	14.2 (27.5)	1.13 (22.2)	5.17 (18.4)
PVH	−9.95 (39.9)	7.73 (37.5)	2.45 (27.2)	0.76 (18.2)

## Data Availability

Partners Healthcare and MGH are not allowed to give access to data without the Principal Investigator (PI) for the human studies protocol first submitting a protocol amendment to request permission to share the data with a specific collaborator on a case-by-case basis. This policy is based on very strict rules dealing with the protection of patient data and information. Anyone wishing to request access to the data must first contact Sarah Derosa, Program Coordinator for Research and Clinical Speech-Language Pathology, Center for Laryngeal Surgery and Voice Rehabilitation, Massachusetts General Hospital: sederosa@partners.org.

## Abbreviations

ACC: Neck Surface Accelerometer
ACFL: AC Flow, i.e., Unsteady Flow Peak-to-Peak Amplitude
ANOVA: Analysis of Variance
FFT: Fast Fourier Transform
FIR: Finite Impulse Response
*f*_0_: Fundamental Frequency
H1–H2: Difference of Magnitudes between First and Second Harmonic
KF: Kalman Filter
MA: Moving Average
MFDR: Maximum Flow Declination Rate
NAQ: Normalized Amplitude Quotient
OVV: Oral Volume Velocity
PVH: Phonotraumatic Vocal Hyperfunction
RMSE: Root-Mean-Square-Error
SNF: Single Notch Filter
VH: Vocal Hyperfunction
